# Coefficient of variation and texture analysis of 18F-FDG PET/CT images for the prediction of outcome in patients with multiple myeloma

**DOI:** 10.1007/s00277-024-05905-7

**Published:** 2024-07-24

**Authors:** Sara Pellegrino, Davide Origlia, Erica Di Donna, Martina Lamagna, Roberta Della Pepa, Fabrizio Pane, Silvana Del Vecchio, Rosa Fonti

**Affiliations:** 1 Department of Advanced Biomedical Sciences, University Federico II, Via Sergio Pansini 5, Naples, 80131 Italy; 2 Department of Clinical Medicine and Surgery, University Federico II, Naples, Italy

**Keywords:** Coefficient of variation, Texture analysis, 18F-FDG PET/CT, Multiple myeloma, Prognosis

## Abstract

In multiple myeloma (MM) bone marrow infiltration by monoclonal plasma cells can occur in both focal and diffuse manner, making staging and prognosis rather difficult. The aim of our study was to test whether texture analysis of 18 F-2-deoxy-d-glucose positron emission tomography/computed tomography (18F-FDG PET/CT) images can predict survival in MM patients. Forty-six patients underwent 18 F-FDG-PET/CT before treatment. We used an automated contouring program for segmenting the hottest focal lesion (FL) and a lumbar vertebra for assessing diffuse bone marrow involvement (DI). Maximum standardized uptake value (SUVmax), mean standardized uptake value (SUVmean) and texture features such as Coefficient of variation (CoV), were obtained from 46 FL and 46 DI. After a mean follow-up of 51 months, 24 patients died of myeloma and were compared to the 22 survivors. At univariate analysis, FL SUVmax (*p* = 0.0453), FL SUVmean (*p* = 0.0463), FL CoV (*p* = 0.0211) and DI SUVmax (*p* = 0.0538) predicted overall survival (OS). At multivariate analysis only FL CoV and DI SUVmax were retained in the model (*p* = 0.0154). By Kaplan-Meier method and log-rank testing, patients with FL CoV below the cut-off had significantly better OS than those with FL CoV above the cut-off (*p* = 0.0003), as well as patients with DI SUVmax below the threshold versus those with DI SUVmax above the threshold (*p* = 0.0006). Combining FL CoV and DI SUVmax by using their respective cut-off values, a statistically significant difference was found between the resulting four survival curves (*p* = 0.0001). Indeed, patients with both FL CoV and DI SUVmax below their respective cut-off values showed the best prognosis. Conventional and texture parameters derived from 18F-FDG PET/CT analysis can predict survival in MM patients by assessing the heterogeneity and aggressiveness of both focal and diffuse infiltration.

## Introduction

Multiple myeloma is a malignant neoplasm characterized by uncontrolled growth of monoclonal plasma cells primarily in the bone marrow. This disease, despite the availability of newer therapies, has a highly variable clinical outcome due to its intrinsic heterogeneous nature at multiple levels including cytogenetic features, biologic characteristics and clinical presentation. In fact, MM can lead to different patterns of bone marrow involvement with the occurrence of focal lesions and/or diffuse infiltration. These peculiarities make staging and prognosis of MM rather difficult [1, 2].

Therefore, various attempts have been made over the years to obtain an accurate staging of this disease either by the introduction of the International Staging System (ISS) and the Revised International Staging System (RISS) [3, 4] either by improving the classical Durie and Salmon staging system with the inclusion of more advanced imaging modalities such as magnetic resonance imaging (MRI) and 18F-FDG PET/CT [5]. In fact, 18 F-FDG PET is now used for initial staging and therapeutic monitoring of MM patients [6]. Furthermore, its prognostic value in this hematologic disease has been widely reported in the literature [7–9]. Indeed, the International Myeloma Working Group issued a consensus statement indicating that 18F-FDG PET/CT, performed at the onset of MM, is a reliable tool for predicting prognosis [10].

Interpretation of 18F-FDG PET/CT images has been, so far, primarily based on visual analysis and on conventional quantitative measurements such as SUVmax. However, more advanced approaches to PET image analysis have been suggested in recent years such as volumetric parameters and texture features [11–14]. The volumetric parameters metabolic tumor tolume (MTV) and total lesion glycolysis (TLG) have been proven to have prognostic significance in MM [15–19]. These parameters are obtained by segmentation of the focal lesions detected by PET by using a predefined threshold. Total MTV and TLG are obtained by summing the metabolic volumes of all focal lesions, thus reflecting the total tumor burden in terms of metabolically active mass of all focal lesions in a MM patient [15]. However, volumetric parameters do not take into account either inter-lesional and intra-lesional heterogeneity [20] or the possible presence of diffuse bone marrow involvement in patients with MM.

In the last years, quantification of intratumor heterogeneity has been performed by texture analysis of images obtained with various imaging methodologies such as computed tomography (CT), MRI and PET/CT mostly in solid tumors such as lung [21–23]. Previous studies on texture analysis of 18F-FDG PET/CT images hypothesized that tumor heterogeneity may be associated with the non-uniform distribution of 18 F-FDG. Spatial distribution of 18 F-FDG, in fact, may be related to a combination of underlying biological processes that may lead to treatment failure and poor overall survival [12, 21, 24, 25]. Few studies have used texture analysis of 18F-FDG PET/CT images for the assessment of intratumor heterogeneity in MM, which can occur in focal lesions as well as in diffuse bone marrow infiltration and could therefore be of importance in the prognosis of this disease [26–29]. Among the numerous texture variables, the Coefficient of Variation (CoV) is a first order parameter derived from standard deviation (SD) divided by SUVmean. This parameter is easy to be determined and provides a synthetic index of the heterogeneity of 18 F-FDG uptake. In particular, our previous studies showed that CoV is an independent prognostic variable of overall survival in lung cancer patients [30] and when combined with CoV of involved lymph nodes may identify lung cancer patients with good or poor prognosis [31].

The aim of our study was, therefore, to test whether CoV and other selected variables derived from texture analysis of 18F-FDG PET/CT images on both focal lesions and diffuse bone marrow infiltration can be of use in predicting survival in MM patients.

## Materials and methods

### Patients

We studied retrospectively the 18F-FDG PET/CT scans of 46 patients (17 females, 29 males; mean age ± SD 63 ± 11 years) using the following inclusion criteria: histopatologically confirmed MM, stage IIIA according to Durie and Salmon staging System, whole-body 18F-FDG PET/CT scan performed at our institution before any therapy and the presence of at least a focal lesion suitable for segmentation (SUVmax > 2.5). The exclusion criteria were presence of prior or concurrent malignancy; previous chemotherapy or radiotherapy; missing imaging data for analysis and missing clinical and imaging follow up. The study was approved by the Institutional Ethics Committee (protocol no. 352/18), and all patients signed an informed consent to undergo PET/CT procedure. Patients clinicopathological characteristics are reported in Table 1.

**Table 1:** Patient characteristics

Characteristics	Median (range)	*N* (%)
Male		29 (63)
Age	66 (32–81)	
Hemoglobin (g/dL)	12.8 (7.0-15.9)	
Plasma cell infiltration (%)	25.0 (1.0–90.0)	
Monoclonal Component (g/dL)	2.0 (0.2–7.6)	
Albumin (g/dL)	4.2 (2.2–5.6)	
b2-microglobuline (mg/dL)	2.3 (1.3–9.3)	
ISS I ISS II ISS III		28 (60) 14 (30) 4 (0.8)

*ISS* International staging system

All patients were subjected to therapeutic regimens with conventional and novel agents followed by autologous bone marrow transplantation in 16 patients. The mean follow-up period was 51 months (range 1-145 months, median 27 months). Overall Survival (OS) was measured from the date of 18F-FDG PET/CT to that of death.

### **18F-FDG PET/CT study**

Whole-body 18F-FDG PET/CT scans were performed 60 min after intravenous administration of 18 F-FDG (370 MBq) in patients who had fasted for the previous 8 h and showing a blood glucose level < 120 mg/dl measured at the time of injection. 18F-FDG PET/CT was performed by using a combined PET/CT Ingenuity TF scanner (Philips Healthcare, Best, The Netherlands). Multidetector CT scan was acquired using the following parameters: 120 kV, 80 mAs, 0.8 s rotation time, pitch of 1.5, PET scan was performed in 3-dimensional mode (3 min per bed position) in caudocranial direction from the feet to the top of the skull. Iterative images reconstruction was performed with an ordered subsets-expectation maximization algorithm. Attenuation corrected emission data were obtained using filtered back projection of CT reconstructed images (Gaussian filter with 8 mm full width half maximum) to match the PET resolution. Transaxial, sagittal, and coronal images as well as coregistered images were preliminary examined using Ingenuity TF software.

### 18 F-FDF PET/CT image analysis

PET/CT data were transferred in digital imaging and communications in medicine (DICOM) format to a workstation and analysed by LIFEx program [32]. Focal lesions were defined as focal areas of increased 18 F-FDG uptake visible on 2 contiguous PET slices at least and not corresponding to physiological tracer uptake or to benign bone pathologies.

**Fig. 1:**
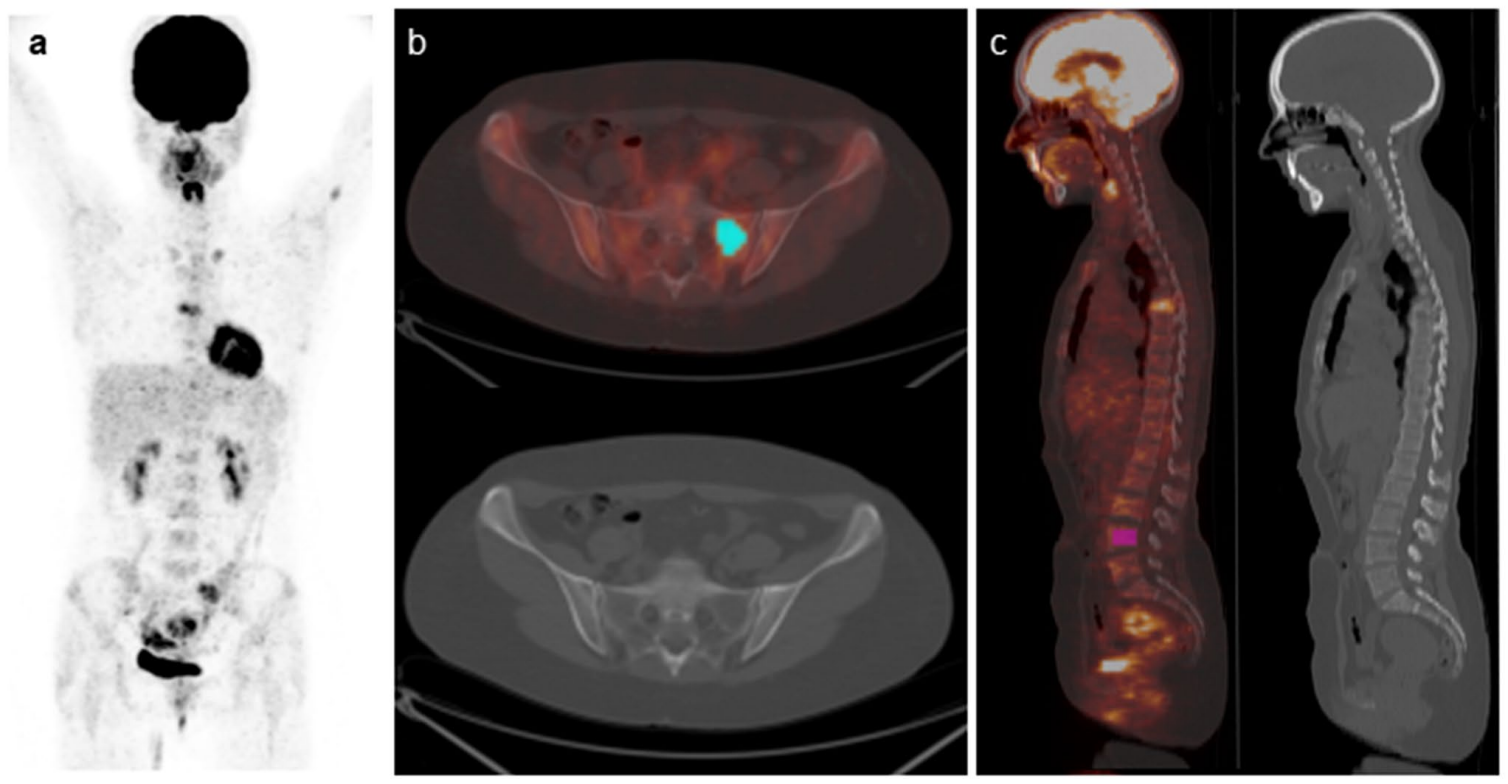
Representative images of 18F-FDG PET/CT scan in a MM patient. (**a**) Maximal intensity projection PET image; (**b**) transaxial fusion images of co-registered PET and CT showing the segmentation (light blue) of the hottest focal lesion; (**c**) sagittal fusion images of co-registered PET and CT showing the segmentation (pink) of a lumbar vertebral body for assessing diffuse bone marrow involvement

A volume of interest (VOI) was delineated on PET images by drawing a tridimensional region around the hottest focal lesion using an automated contouring program setting an absolute threshold for SUV at 2.5 in accordance with previous studies [11, 33, 34]. In addition, the accuracy of lesion delimitation was confirmed on the corresponding CT images. Diffuse bone marrow involvement was also measured within a VOI obtained by drawing a tridimensional region of interest (4 cm^3^) inside the body of a lumbar vertebra (preferably L2 to L4) not showing focal uptake. By computed analysis of each VOI, 46 features (39 texture features and 7 conventional parameters) were extracted. Among all these variables, we selected texture features that, in previous studies [21, 25, 35] had shown sufficient robustness and repeatability as well as significant correlation with clinical outcome in cancer patients. Therefore, along with the conventional imaging parameters SUVmax and SUVmean, we selected: 3 first order features such as CoV, (Standard Deviation divided by SUVmean), histogram (HISTO) Skewness and HISTO Kurtosis and 3 features of higher order such as gray level co-occurrence matrix (GLCM) Entropy(log_10_), GLCM Dissimilarity and Neighboring gray-level dependence matrix (NGLDM) Coarseness. Representative images of VOIs drawn around a focal lesion or depicting diffuse bone marrow on a lumbar vertebral body are shown in Fig. 1.

### Statistical analysis

Statistical analysis was performed using the software MedCalc for Windows, version 10.3.2.0 (MedCalc Software, Mariakerke, Belgium). A probability value of < 0.05 was considered statistically significant. Student’s t-test was used to compare means of unpaired data. The Pearson’s correlation coefficient was used to evaluate the linear relationship between continuous variables. Univariate and multivariate analysis of imaging variables were performed using Cox proportional hazards regression. Variables that predicted OS by univariate analysis were included in the model for multivariate analysis. Receiver operating characteristic (ROC) curve analysis was performed to estimate the best discriminative value of independent prognostic variables between dead and survivors. Survival analysis was performed using the Kaplan-Meier method and log-rank tests. Survivors were censored at the time of the last clinical control.

## Results

18F-FDG PET/CT scans of 46 MM patients were studied. The hottest focal lesion (FL) and the diffuse involvement (DI) of the bone marrow measured on a lumbar vertebra were analyzed in each patient. In particular, conventional imaging parameters such as SUVmax and SUVmean along with the first order texture variable CoV were obtained from 46 focal lesions and 46 lumbar vertebral bodies. Moreover, other 2 first order texture variables (HISTO Skewness, HISTO Kurtosis) and 3 higher order features [GLCM Entropy(log_10_), GLCM Dissimilarity, NGLDM Coarseness] were extracted from the analysis of 38 focal lesions, as lesions with VOI ≤ 64 voxels were too small to be included in texture analysis, and from all 46 lumbar vertebrae. Mean ± SD and range values of the all variables obtained from both FL and DI namely: SUVmax, SUVmean, CoV, HISTO Skewness, HISTO Kurtosis, GLCM Entropy(log_10_), GLCM Dissimilarity and NGLDM Coarseness are reported in Table 2.

**Table 2:** Mean, median and range values of conventional imaging parameters and texture features obtained by 18 F-FDG PET/TC analysis of the hottest focal lesion and diffuse bone marrow involvement

Variables	Mean ± SD	Range
SUVmax		
FL DI	11.05 ± 8.30 3.00 ± 0.96	2.80-42.01 1.31–5.43
SUVmean		
FL DI	4.89 ± 2.21 1.80 ± 0.57	2.19–11.77 0.71–3.04
CoV		
FL DI	0.35 ± 0.15 0.23 ± 0.41	0.12–0.67 0.16–0.34
HISTO Kurtosis		
FL DI	3.45 ± 1.21 3.10 ± 0.78	1.71–6.27 2.06–5.79
HISTO Skewness		
FL DI	0.96 ± 0.43 0.34 ± 0.41	0.27–1.74 -0.60-1.62
GLCM Entropy (log_10_)		
FL DI	1.99 ± 0.55 1.13 ± 0.22	1.09–3.11 0.68–1.65
GLCM Dissimilarity		
FL DI	4.29 ± 2.73 0.96 ± 0.31	1.13–11.18 0.49–1.90
NGLDM Coarseness		
FL DI	0.02 ± 0.01 0.06 ± 0.09	0.002–0.072 0.04–0.08

*SD* Standard Deviation, *SUVmax* Maximum Standardized Uptake Value, *FL* hottest Focal Lesion, *DI* Diffuse Involvement, *SUVmean* Mean Standardized Uptake Value, *CoV* Coefficient of Variation, *GLCM* Gray-Level Co-occurrence Matrix, *NGLDM* Neighborhood Gray-Level Dependence Matrix

As expected, the mean values of all variables were higher in FL than in DI especially SUVmax (11.05 ± 8.30 vs. 3.00 ± 0.96), SUVmean (4.89 ± 2.21 vs. 1.80 ± 0.57) and GLCM Dissimilarity (4.29 ± 2.73 vs. 0.96 ± 0.31). This latter variable extracted from FL was negatively and significantly correlated with the amount of monoclonal component (*r*=-0.4037, *p* = 0.0453) and almost significantly with the percentage of plasma cell infiltration (*r*=-0.3528, *p* = 0.0605). Moreover, among the variables obtained from DI measurement, SUVmax and SUVmean correlated with plasma cell infiltration (*r* = 0.6683, *p* < 0.0001 and *r* = 0.6524, *p* < 0.0001, respectively), monoclonal component (*r* = 0.422, *p* = 0.018 and *r* = 0.4351, *p* = 0.0144, respectively) and inversely with hemoglobin (*r*=-0.3258, *p* = 0.029 and *r*=-0.3059, *p* = 0.041, respectively), GLCM Entropy(log_10_), GLCM Dissimilarity and NGLDM Coarseness correlated with plasma cell infiltration (*r* = 0.6383, *p* = 0.0001, *r* = 0.5172, *p* = 0.0021 and *r* = 0.3649, *p* = 0.0368, respectively), while CoV significantly correlated with albumin levels (*r* = 0.3235, *p* = 0.0322).

After a mean follow-up period of 51 months, 24 patients died from MM and were compared to the 22 survivors for OS analysis. Univariate analysis showed that among the conventional and texture variables obtained from FL, SUVmax (χ^2^ = 4.006, *p* = 0.0453), SUVmean (χ^2^ = 3.97, *p* = 0.0463) and CoV (χ^2^ = 5.32, *p* = 0.0211) predicted OS, while GLCM Entropy(log_10_) (χ^2^ = 3.423, *p* = 0.0643) and GLCM Dissimilarity (χ^2^ = 3.359, *p* = 0.0668) were almost predictive of OS. Moreover, among the variables obtained from DI, SUVmax only predicted OS (χ^2^ = 3.718, *p* = 0.0538) while SUVmean (χ^2^ = 3.37, *p* = 0.0664) was almost predictive of OS (Table 3).

**Table 3:** Predictors of overall survival by univariate analysis based on conventional and texture imaging parameters obtained from focal lesions and diffuse bone marrow involvement

Variables	χ^2^	*P*
SUVmax		
FL DI	4.006 3.718	0.0453 0.0538
SUVmean		
FL DI	3.97 3.37	0.0463 0.0664
CoV		
FL DI	5.32 0.0864	0.0211 0.7688
HISTO Kurtosis		
FL DI	1.252 0.0000472	0.2631 0.9945
HISTO Skewness		
FL DI	0.584 0.331	0.4449 0.5653
GLCM Entropy (log_10_)		
FL DI	3.423 2.51	0.0643 0.1131
GLCM Dissimilarity		
FL DI	3.359 2.939	0.0668 0.0864
NGLDM Coarseness		
FL DI	2.79 0.00477	0.0948 0.9449

*SUVmax* Maximum Standardized Uptake Value, *FL* hottest Focal Lesion, *DI* Diffuse Involvement, *SUVmean* Mean Standardized Uptake Value, *CoV* Coefficient of Variation, *GLCM* Gray-Level Co-occurrence Matrix, *NGLDM* Neighborhood Gray-Level Dependence Matrix

**Fig. 2:**
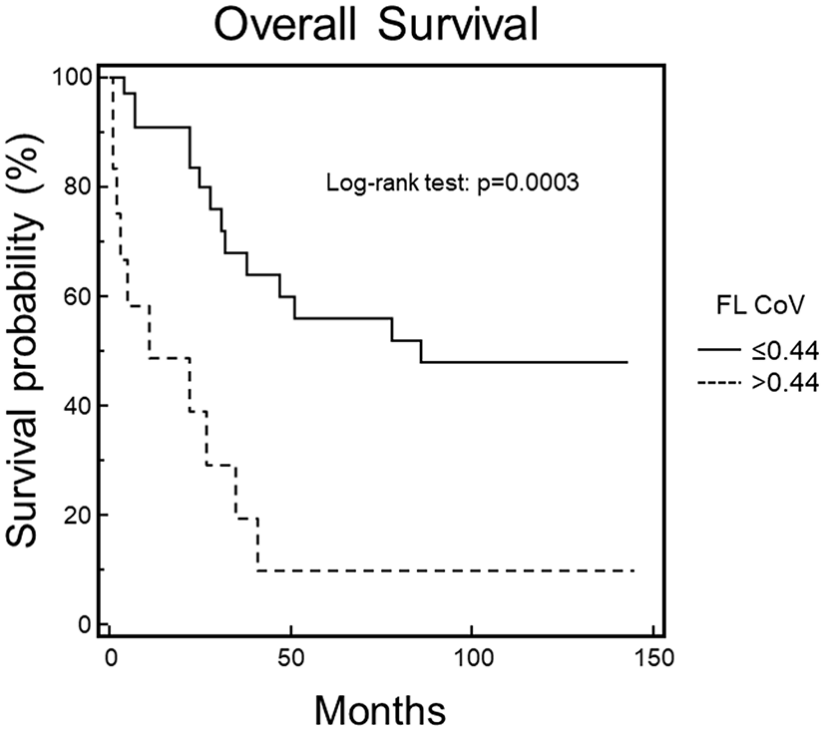
Overall survival by Kaplan-Meier analysis and log-rank test at 51-month follow-up. Statistically significant difference in OS between MM patients with FL CoV values lower or higher than the cut-off level of 0.44, as assessed by ROC curve analysis (*p* = 0.0003)

When these variables were entered in the multiple regression model only FL CoV and DI SUVmax were retained in the model (χ^2^ = 8.344, *p* = 0.0154). ROC curve analysis showed that the best FL CoV and DI SUVmax values discriminating between survivors and patients who had died were 0.44 (AUC 0.659) and 3.88 (AUC 0.608), respectively. OS curve estimated by Kaplan-Meier method and log-rank test, in fact, was significantly prolonged in patients with FL CoV ≤ 0.44 as compared with that of patients with FL CoV > 0.44 (χ^2^ = 13.2135, *p* = 0.0003) (Fig. 2).

**Fig. 3:**
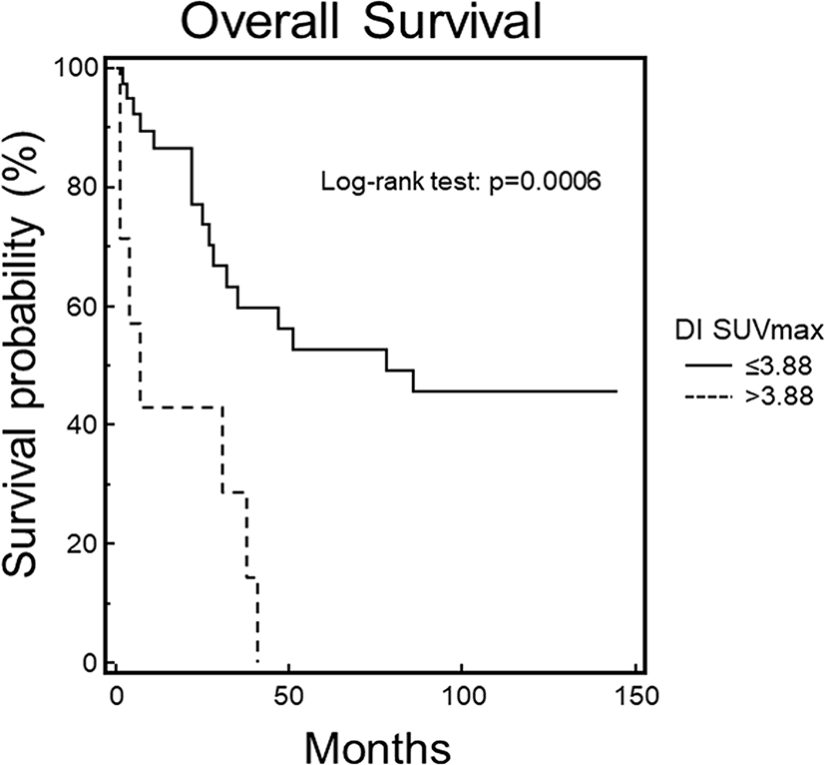
Overall survival by Kaplan-Meier analysis and log-rank test at 51-month follow-up. Statistically significant difference in OS between MM patients with DI SUVmax values lower or higher than the cut-off level of 3.88, as assessed by ROC curve analysis (*p* = 0.0006)

Similarly, patients with DI SUVmax ≤ 3.88 had a significantly better OS than those with DI SUVmax > 3.88 (χ^2^ = 11.8658, *p* = 0.0006) (Fig. 3).

**Fig. 4:**
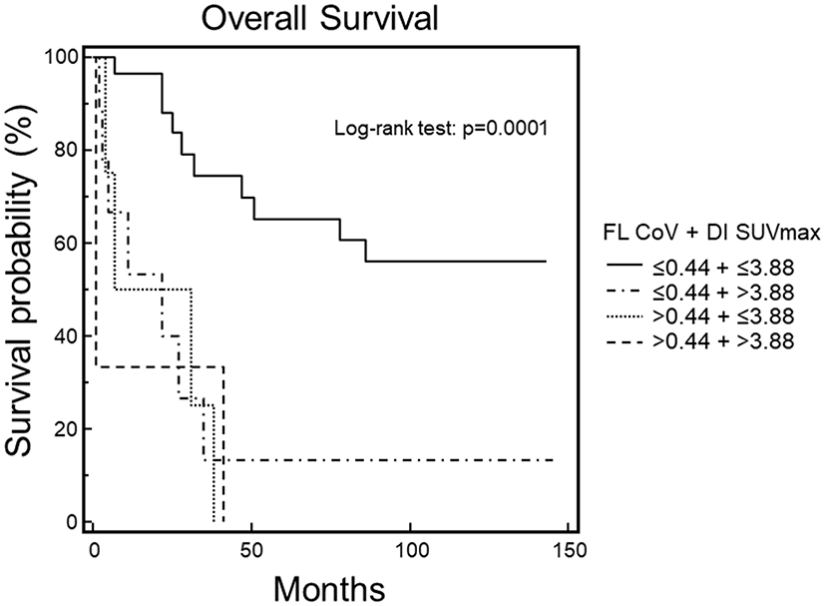
Overall survival by Kaplan-Meier analysis and log-rank test using all possible combinations of FL CoV and DI SUVmax. A statistically significant difference was found among the four survival curves (*p* = 0.0001). In particular, patients with both FL CoV and DI SUVmax below their respective cut-off values showed the best prognosis

Finally, combining FL CoV and DI SUVmax for Kaplan-Meyer analysis by using the respective cut-off values, we found a statistically significant difference among the four resulting survival curves (χ^2^ = 20.8005, *p* = 0.0001). In fact, patients with FL CoV ≤ 0.44 and DI SUVmax ≤ 3.88 had the most favorable prognosis, as opposed to those with FL CoV and DI SUVmax higher than their respective cut-off values. Moreover, the other two subgroups of patients showed an intermediate survival (Fig. 4).

## Discussion

Our study showed that Coefficient of Variation of focal lesions and SUVmax of bone marrow both extracted by texture analysis of 18F-FDG PET/CT images are independent predictive factors of clinical outcome in MM patients. In particular, patients with focal lesions showing CoV values higher than the cut-off had significantly worse prognosis as compared to patients with focal lesions having CoV values lower than the threshold. Similarly, the intensity of diffuse bone marrow uptake predicted good or poor prognosis in the groups of patients having SUVmax lower or higher than the threshold, respectively. Notably, CoV of focal lesions combined with SUVmax of bone marrow were able to identify the group of patients with the best prognosis characterized by values of both parameters below the correspondent threshold.

The biologic meaning of texture features including CoV still needs to be clarified. In MM it could be of aid in understanding the underlying aspects of tumor biology related to clonal plasma cells or cell interactions. In this regard, the finding that OS was significantly worse in patients with CoV higher than the cut-off may be explained by the fact that heterogeneity of FDG uptake in focal lesions is mainly due to the invasion of bone marrow by clonal plasma cells having a glycolytic phenotype. CoV and other features extracted by texture analysis of 18F-FDG PET/CT images have been tested in the diagnosis, therapy response assessment and prognosis of various solid tumors [12, 25, 30, 31, 36–40] and lymphoproliferative diseases including MM [41–43]. In particular, in MM, numerous first and higher order texture features processed by different Machine Learning algorithms have been tested in the attempt to improve the prognostic stratification of these patients. Some studies [26–28] found that the predictive model which included both PET/CT based texture features and conventional clinical variables showed the best performance in predicting prognosis of MM patients. Furthermore, among the different Machine Learning methods tested, Random Survival Forest showed to be the most suitable model for predicting progression-free survival (PFS) in MM [28, 29].

Diffuse bone marrow involvement is an important component of MM in several patients contributing to the severity of this heterogeneous disease. Evaluation of diffuse bone marrow involvement on PET images has been usually performed by visual assessment also using Deauville score [44, 45]. In previous studies, qualitatively assessed bone marrow involvement was not predictive of survival [17, 46, 47]. However, in a recent study [29] bone marrow SUVmax obtained by texture analysis was the only imaging feature predictive of PFS in patients with MM. In another recent study [48] bone marrow SUVmax along with other texture features was able to assess the presence of minimal residual disease after treatment in MM patients.

In relation to the other selected variables of texture analysis, they showed significant correlations with classical biochemical and histopathological factors routinely used for staging MM patients. In particular, dissimilarity, a second order feature of texture analysis that describes the local variation of the grey level of voxel pairs in an image [30], when obtained by focal lesions, was almost predictive of OS at univariate analysis even though statistical significance was not achieved due to the limited number of observations. In fact, the higher the dissimilarity in focal lesions, the worse the prognosis. Nevertheless, dissimilarity was negatively correlated with the amount of monoclonal component and with the percentage of plasma cell infiltration in the bone marrow. This could mean that the higher the dissimilarity in focal lesions, the lower the number of multiple myeloma cells producing monoclonal component in the bone marrow. Notably, when dissimilarity, coarseness and entropy were determined on diffuse bone marrow uptake, they showed a direct significant correlation with plasma cell infiltration and were not predictive of OS at univariate analysis. These observations provide several clues to understand the biological meaning of texture variables that are often difficult to interpret.

In conclusion, our study shows that texture features extracted by 18F-FDG PET/CT analysis can be of aid in the prediction of clinical outcome of MM patients by providing information on both focal and diffuse infiltration by clonal plasma cells. In particular, CoV, by indicating the heterogeneity of clonal cell distribution within focal lesions and SUVmax, by reflecting the aggressiveness of diffuse bone marrow infiltration were both independent predictors of clinical outcome and when combined improved the risk stratification of MM patients. Furthermore, both CoV and SUVmax are simple first order texture features, easy to calculate, having the advantage that their extraction from 18F-FDG PET/CT images does not require the use of sophisticated software.

## Data Availability

The data that support the findings of this study are available on request from the corresponding author.
